# HTLV-1 and Co-infections

**DOI:** 10.3389/fmed.2022.812016

**Published:** 2022-02-03

**Authors:** Carolina Rosadas, Graham P. Taylor

**Affiliations:** ^1^Section of Virology, Department of Infectious Disease, Imperial College London, London, United Kingdom; ^2^National Centre for Human Retrovirology, Division of Medicine and Integrated Care, St. Mary's Hospital, Imperial College Healthcare NHS Trust, London, United Kingdom

**Keywords:** HTLV-1, co-infection, *Mycobacterium tuberculosis*, *Mycobacterium leprae*, HCV, HBV, sexually transmitted infections, *Schistosoma mansoni*

## Abstract

Human T lymphotropic virus type 1 (HTLV-1) is a retrovirus that causes lifelong T-cell infection in humans, impacting the host immune response. This virus causes a range of clinical manifestations, from inflammatory conditions, including neuronal damage (HTLV-1 associated myelopathy, HAM) to life-threatening leukemia (adult T-cell leukemia, ATL). Human T lymphotropic virus type 1 is also associated with increased risk of all-cause mortality, but the mechanisms remain unclear. As a blood-borne and sexually transmitted infection (STI), HTLV-1 shares transmission routes to many other pathogens and although it has worldwide distribution, it affects mainly those in low- and middle-income tropical areas, where the prevalence of other infectious agents is high. These factors contribute to a high incidence of co-infections in people living with HTLV-1 (PLHTLV). This comprehensive review addresses the impact of HTLV-1 on several co-infections and vice-versa. There is evidence of higher rates of HTLV-1 infection in association with other blood borne (HCV, HBV) and sexually transmitted (Syphilis, Chlamydia, HPV, HSV) infections but whether this represents increased susceptibility or opportunity is unclear. Higher frequency of *Mycobacterium tuberculosis (MTb)* and *Mycobacterium leprae (M. leprae)* is observed in PLHTLV. Reports of opportunistic infections and high frequency of crusted scabies in patients with HTLV-1 points to immune impairment in those individuals. Human T lymphotropic virus type 1 may influence the persistence of pathogens, exemplified by the higher rates of *Schistosoma mansoni* and *Strongyloides stercoralis (St. stercoralis)* treatment failure observed in PLHTLV. This retrovirus is also associated with increased tuberculosis (TB) severity with some evidence pointing to a deleterious impact on leprosy outcome as well. These findings are supported by immune alterations observed in those co-infected individuals. Although the role of HTLV-1 in HCV outcome is debatable, most data indicate that HTLV may negatively impact the clinical course of hepatitis C. Co-infections may also influence the risk of developing HTLV-1 associated disease, but data are still limited. The impact of HTLV-1 on the response to more common infections, might contribute to the increased mortality rate of HTLV-1. Large scale prospective controlled studies on the prevalence and impact of HTLV-1 in co-infections and vice-versa are needed. Human T lymphotropic virus type 1 impact in public health is broad. Measures to increase awareness and to prevent new infections are needed.

## Introduction

Human T lymphotropic virus type 1 (HTLV-1) causes lifelong infection mainly in CD4^+^ cells. The immune imbalance that occurs in HTLV-1 infection is linked to the pathogenesis of HTLV-1 associated diseases. The neurological disease HTLV-1 associated myelopathy/tropical spastic paraparesis (HAM/TSP), that greatly impacts patient‘s quality of life and can result in wheelchair dependency, and other conditions, such as uveitis and thyroiditis are examples of the many inflammatory manifestations associated with HTLV-1. Adult T-cell leukemia/lymphoma (ATL), the usually fatal neoplasm caused by HTLV-1 is also linked to immunological impairment as well as monoclonal expansion and transformation of an infected T-cell. There are several evidences that patients with ATL have severe immunosuppression which is associated with opportunistic infections and other malignancies (1–4). However, even in the absence of clinical disease, HTLV-1 can cause immune impairment (2, 5). Unprotected sexual intercourse, contact with blood or tissue and breastfeeding may expose individuals not only to HTLV-1 but to a variety of other pathogens, such as human immunodeficiency virus (HIV), *Treponema pallidum*, hepatitis C virus (HCV), hepatitis B virus (HBV), human papillomavirus (HPV), and herpes simplex virus (HSV). Moreover, the distribution of HTLV-1, with high prevalence observed mainly in low- and middle-income countries (LMIC) (6), overlaps with areas where the prevalence of other infections, such as *Strongyloides stercoralis (St. stercoralis), Mycobacterium tuberculosis (MTb)*, and *Mycobacterium leprae (M. leprae)*, is high. Thus, HTLV-1 infected individuals can be co-infected with a range of pathogens. Indeed, the prevalence of co-infection is high in some settings. The interactions between coexisting pathogens are complex and may affect the natural course of both infections. While an anti-viral immune response involves a dominant Th1 type response, the ideal response to some parasites, e.g., helminths, usually involves a dominant Type-2 response. Therefore, the response to co-infections can be conflicting from an immunological perspective (5). Furthermore, clinical conditions associated with different pathogens, including HTLV-1 itself, may involve an exacerbated response in one arm of the immunological system. Thus, co-infections can be harmful or occasionally even beneficial to the host. Recently, a meta-analysis showed that HTLV-1 infection is associated with increased risk of death and identified 16 clinical conditions associated with, or occurring more frequently with, HTLV-1 infection [seborrheic dermatitis, Sjogren's syndrome, eczema, pulmonary alteration, asthma, fibromyalgia, rheumatoid arthritis, arthritis, tuberculosis (TB), kidney and bladder infections, dermatophytosis, community acquired pneumonia, strongyloides hyperinfection syndrome, liver cancer; lymphoma other than ATL, and cervical cancer] (7). However, the range of reported disease associations is broader still and here a number of questions concerning the impact of HTLV-1 infection on the susceptibility and outcome of other infections and vice-versa are addressed.

## Do Other Infections Increase the Likelihood of HTLV-1 Co-infection or Vice-Versa?

### Mycobacteria

In a Japanese study from the 80's Hanada et al. demonstrated a higher prevalence of HTLV-1 infection among patients with pulmonary TB when compared to healthy individuals [29.5% (64/217) vs. 11.9% (562/4,741), *p* < 0.01] but confirmatory tests for HTLV-1 infection were not performed (8). Also in Japan, the prevalence of anti-HTLV-1 antibodies was higher in patients with TB than in a control group with bronchial asthma (17/105 vs. 4/58, OR = 2.61, 95% CI 0.83–8.16) (9). In a case-control study of 375 patients with TB and 378 control subjects in Brazil, HTLV-1 prevalence was higher in TB patients (4.3%, 16/375) than in a control group (1.3%, 5/378), resulting in a crude OR 3.31 (95% CI, 1.20–9.13) that remained statistically significant after controlling for numerous variables (age, sex, education, income, ethnicity, sexual history, history of blood transfusion, and intravenous drug use) (10). Similar results were observed in two other cross-sectional studies in Brazil (10.9 and 11.1% HTLV-1 infection in TB patients vs. 4.5 and 1.8% in control patients without TB, respectively) (11, 12). In the same country, the prevalence of HTLV-1 infection in patients with TB was higher than in pregnant women (2/451 vs. 1/814, OR = 3.62, 95% CI 0.33–40.05) (13). In Bissau, although the prevalence of HTLV-1 infection was higher in TB patients when compared to a population-based control group [11.4% (32/280) vs. 3.5% (74/2, 117), OR = 3.6 (95% CI = 2.2–5.6), *p* < 0.05], the results were not statistically significant when separately evaluating HIV-negative TB patients [3.7% (6/162) vs. 2.6% (51/1,930), OR = 1.18 (95% CI = 0.48–2.89), *p* = 0.71] (14). The authors hypothesized that the immunosuppression caused by HTLV-1 alone was insufficient to increase the risk of TB, but it adds to the risk of TB among HIV-infected individuals. Later, the same group observed higher mortality in patients with pulmonary TB when coinfected with HIV-2/HTLV-1 compared to HIV-2-positive HTLV-1-negative patients in Guinea-Bissau (15). There was no statistically significant difference between HTLV-1 prevalence in patients with TB comparing to general population from Ivory Coast [1/41 (2.4%) vs. 23/1,291 (1.8%)] (16) and Senegal [3/197(1.5%) vs. 2/181 (1.1%)] (Table 1) (19).

**Table 1:** Prevalence of HTLV-1 infection in patients with *M. tuberculosis*.

**Study**	**Sample size**	**Prevalence (%)**	**95% CI**	**Weight (%)**
Seaton (17) (Papua New Guinea)	102	0.0	0–3.5	6.8
Broutet et al. (13) (Brazil)	451	0.4	0.1–1.6	6.4
Kozlowski et al. (18) (Brazil)	402	1.0	0.3–2.5	7.5
Kaplan et al. (19) (Senegal)	197	1.5	0.3–4.4	7.3
Berini et al. (20) (Argentina)	187	2.1	0.6–5.4	7.3
Verdier et al. (16) (Ivory Coast)	41	2.4	0.1–12.8	5.8
Marinho et al. (10) (Brazil)	375	4.3	2.5–6.8	7.5
Pedral-Sampaio et al. (21) (Brazil)	378	8.5	5.9–11.7	7.5
Bastos et al. (22) (Brazil)	360	10.8	7.8–14.5	7.5
Moreira et al. (12) (Brazil)	90	11.1	5.5–19.5	6.8
Norrgren et al. (14) (Guinea-Bissau)	280	11.4	7.9–15.7	7.4
Kohno et al. (9) (Japan)	105	16.2	9.7–24.6	6.9
Grassi et al. (23) (Brazil)	73	45.2	33.5–57.3	6.9
**Total (random effects)**	**3,493**	**8.5**	**5–12.9**	**100.0**

A study conducted in Japan evaluated different diseases among men with HTLV-1 infection and showed that 6.1% (17/278) of HTLV-1 patients had a history of TB compared with 2.9% (74/2,569) of uninfected patients (OR = 3.1 95% CI 1.1–3.3, *p* < 0.05) (24). When analyzing the incidence of TB amongst HTLV-1 infected individuals from Brazil, by cross-matching of records on the Brazilian national registry and the database of the referral center for HTLV, Grassi et al. reported a relative risk of developing TB in HTLV-1 infected patients of 2.6 (CI 95% 1.6–4.2) when compared to the HTLV-1 uninfected group (23). No differences in clinical presentation or TB outcome were observed. In Peru, the history of active TB was analyzed in relatives of HTLV-1 infected patients. History of active TB was more frequent (11.4%, 45/394) among HTLV-1 infected compared to HTLV-1 uninfected relatives (4.3%, 36/839, *p* < 0.001). In a multivariate analysis HTLV-1 infection was associated with TB history in Peru (Adjusted OR 2.5, 95% CI 1.6–3.9) (25). This was also observed in the USA (OR = 2.4, 95% CI 0.82–7.02) (26).

Higher prevalence of HTLV-1 infection among patients with leprosy has been reported from Japan, Democratic Republic of Congo, Congo, and Ivory Coast (8, 16, 27–29) compared to healthy residents, including blood donors (Japan) and the general population (Democratic Republic of Congo, Congo, and Ivory Coast). Similar results were observed even in patients with leprosy from HTLV-1 non-endemic areas from Japan (30). However, other studies found no significant difference regarding HTLV-1 prevalence in patients with *M. leprae* from Yemen, Senegal, Ethiopia, and a non-endemic area for HTLV-1 from Brazil (Curitiba). However, the sample size in each setting was small (27, 31, 32). Prevalence studies are summarized in Table 2 (32, 33, 35).

**Table 2:** Prevalence of HTLV-1 infection in patients with *M. leprae*.

**Study**	**Sample size**	**Prevalence (%)**	**95% CI**	**Weight (%)**
Verdier et al. (27) (Yemen)	117	0.0	0.0–3.1	7.6
Braga et al. (31) (Brazil non endemic area)	199	0.0	0–1.8	7.8
Tekle-Haimanot et al. (32) (Ethiopia)	250	0.4	0–2.2	7.9
Verdier et al. (27) (Senegal)	227	0.9	0.1–3.1	7.8
Glaser et al. (33) (USA)	107	1.9	0.3–6.6	7.5
Machado et al. (34) (Brazil)	245	2.4	0.9–5.2	7.8
Muneishi et al. (30) (Japan)	844	8.5	6.7–10.6	8.0
Kashala et al. (29) (Democratic Republic of Congo)	57	8.8	2.9–19.3	7.1
Milanga et al. (35) (Democratic Republic of Congo)	57	8.8	2.9–19.3	7.1
Verdier et al. (27) (Ivory Coast)	319	9.7	6.7–13.5	7.9
Verdier et al. (27) (Congo)	830	9.9	7.9–12.1	8.0
Verdier et al. (16) (Ivory Coast)	109	13.8	7.9–21.7	7.5
Lechat et al. (28) (Democratic Republic of Congo)	377	37.4	32.5–42.5	7.9
**Total (random effects)**	**3,738**	**5.9**	**2.2**–**11.2**	**100.0**

*Test for heterogeneity: Q = 394.1; DF = 12; p < 0.0001; I^2^ (95% CI) = 96.96% (95.9–97.7)*.

**Figure 1:**
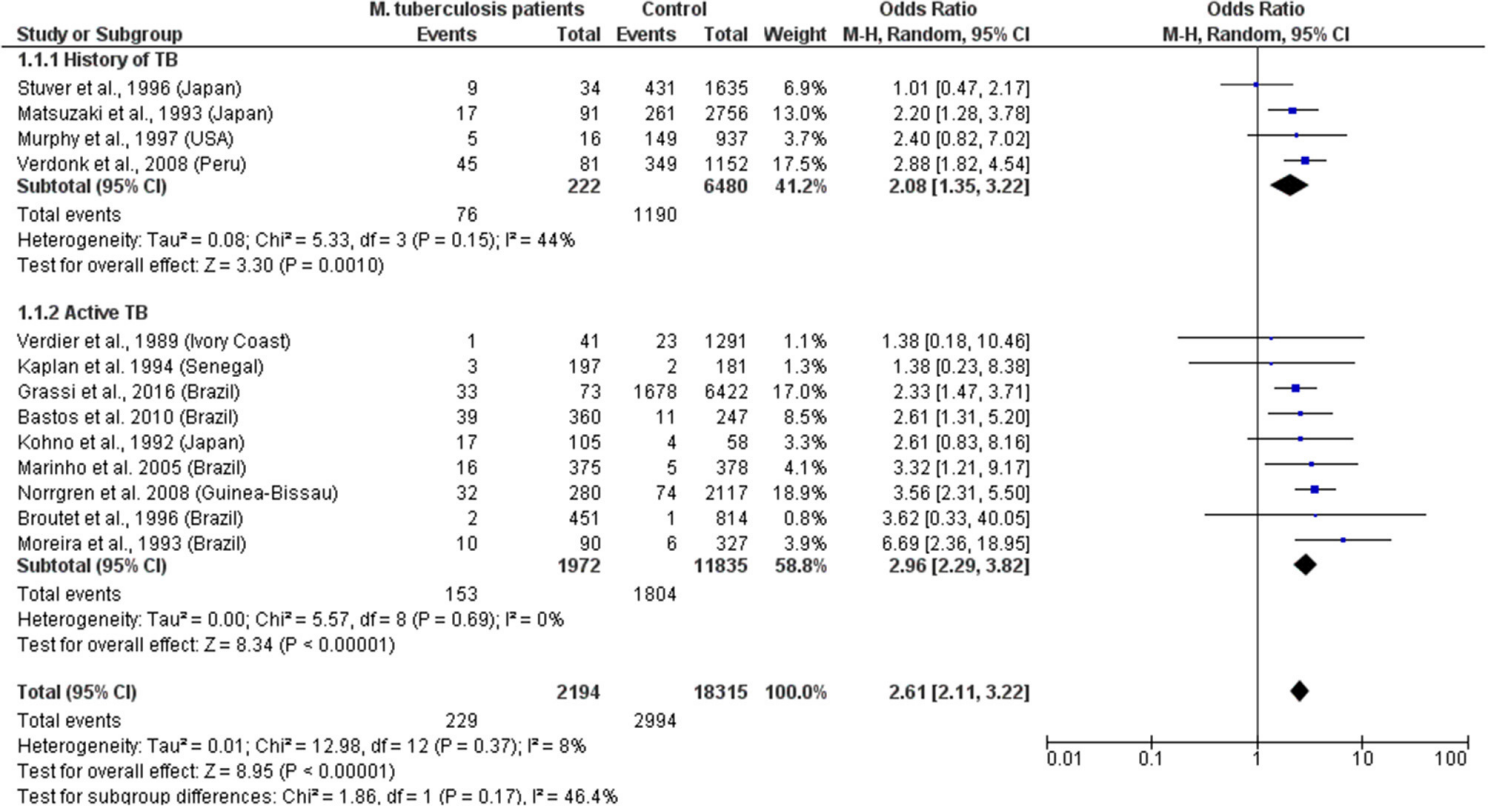
Forest Plot and meta-analysis showing the Odds Ratio of HTLV-1 infection in patients with *Mycobacterium tuberculosis*. The boxes and lines indicate the odds ratios (ORs) and their confidence intervals (CIs) for each study. The pooled odds ratio is represented by a black diamond. The size of the blue squares indicates the relative weight of each estimate. Statistical analysis and graph were performed using RevMan 5 Software. Odds Ratio was calculated using Random effects model and Mantel-Haenszel statistical method. Measures of heterogeneity between studies in each subgroup (those with History of TB and those with Active TB) and between subgroups is shown.

**Figure 2:**
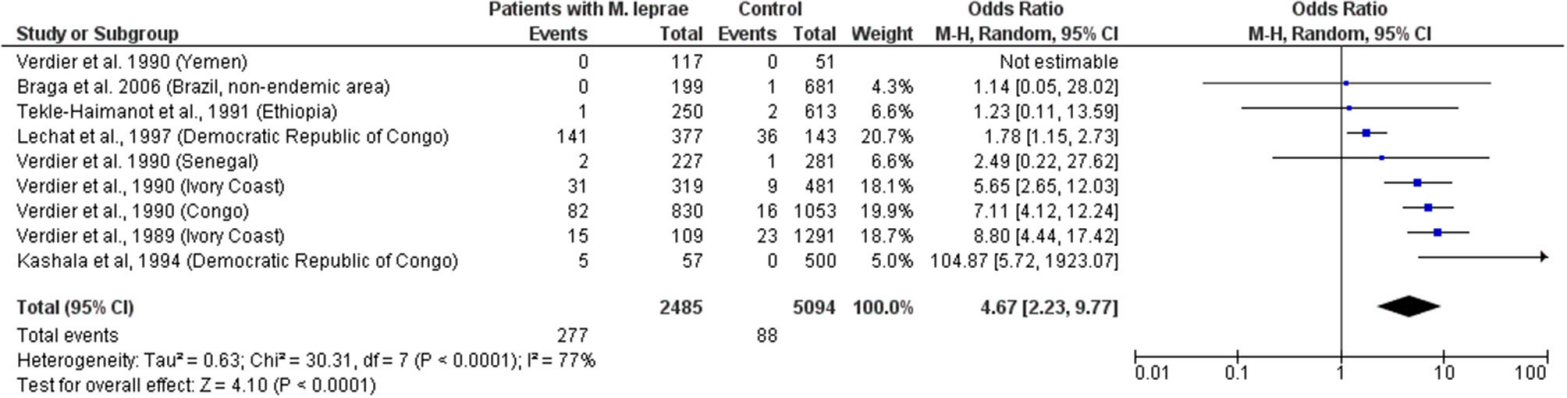
Forest Plot and meta-analysis showing the Odds Ratio of HTLV-1 infection in patients with *Mycobaterium leprae*. The boxes and lines indicate the odds ratios (ORs) and their confidence intervals (CIs) for each study. The pooled odds ratio is represented by a black diamond. The size of the blue squares indicates the relative weight of each estimate. Statistical analysis and graph were performed using RevMan 5 Software. Odds Ratio was calculated using Random effects model and Mantel-Haenszel statistical method. Measures of heterogeneity between studies is shown.

The data indicate that HTLV-1 is more common in patients with *MTb* than expected (combined OR = 2.6, 95% CI 2.1–3.2) suggesting an increased susceptibility to develop TB if HTLV-1 infected or vice versa, the first option being most biologically plausible (Table 1; Figure 1). While patients with a history of TB were twice as likely to have HTLV-1 infection, those with active TB had three times more chance of being co-infected with HTLV-1. The low heterogeneity observed between this subgroup strengthens this finding. Although limited, current data suggests an association between HTLV-1 and *M. leprae* (Pooled OR = 4.67, 95% CI 2.23–9.77) (Table 2; Figure 2), which would fit with the association between HTLV-1 and *MTb*.

### Sexually Transmitted Infections

**Figure 3:**
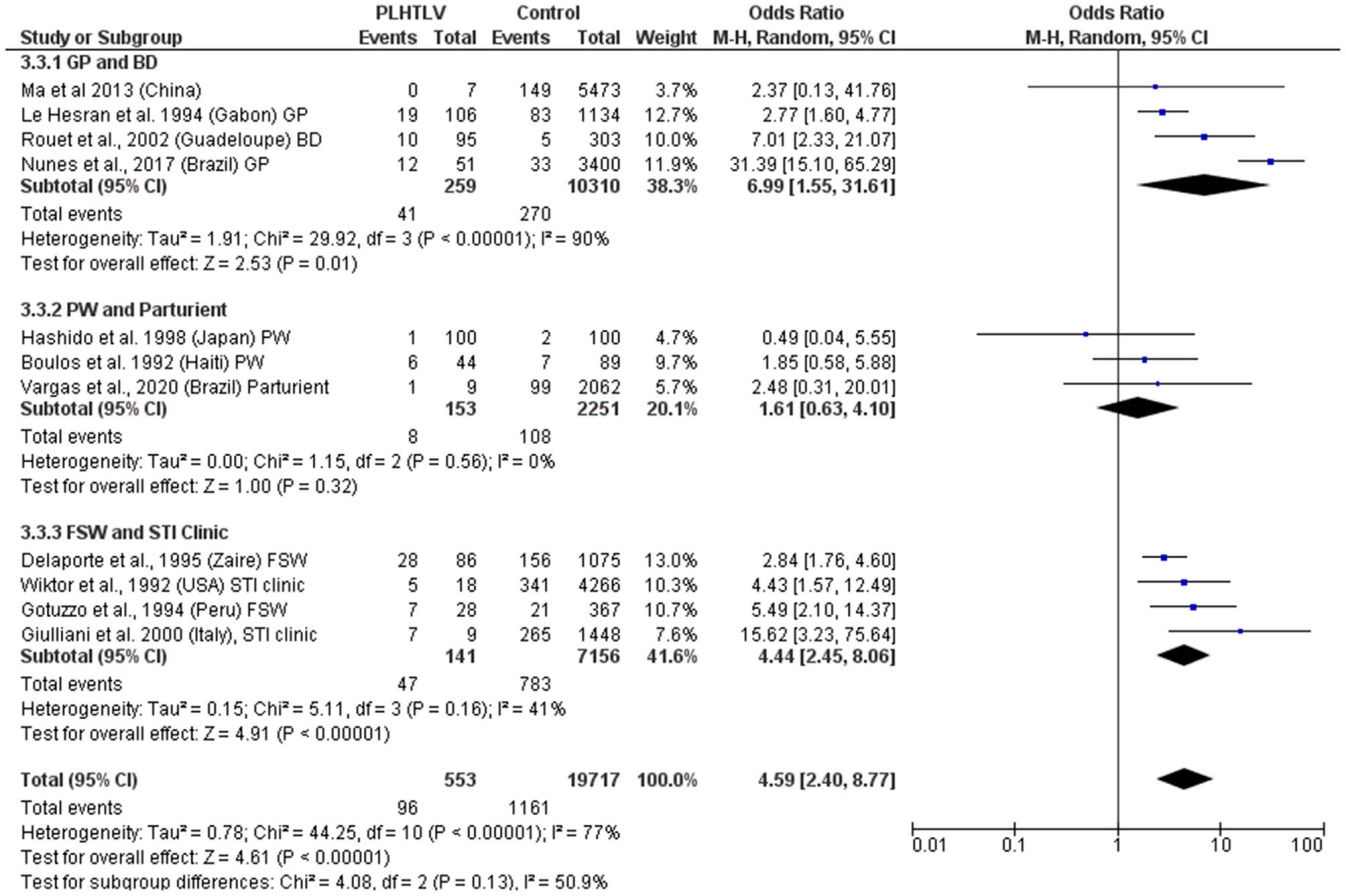
Forest Plot and meta-analysis showing the Odds Ratio of Syphilis infection in people living with HTLV-1. The boxes and lines indicate the odds ratios (ORs) and their confidence intervals (CIs) for each study. The pooled odds ratio is represented by a black diamond. The size of the blue squares indicates the relative weight of each estimate. Statistical analysis and graph were performed using RevMan 5 Software. Odds Ratio was calculated using Random effects model and Mantel-Haenszel statistical method. Measures of heterogeneity between studies in each subgroup (population under investigation) and between subgroups is shown. PLHTLV, people living with HTLV; GP, general population; BD, blood donors; PW, pregnant women; FSW, female sex workers; STI, sexually transmitted infection.

Murphy et al. demonstrated current diagnosis of syphilis to be an independent risk factor for HTLV-1 infection in both females [OR = 2.12 (1.12–3.99)] and males [OR = 3.56 (1.24–10.22)] and hypothesized that although the efficiency of HTLV-1 sexual transmission is lower from female-to-male, it can be increased by the presence of a penile ulcer or concurrent syphilis (36). In the US, a history of syphilis (OR = 4.2, *p* = 0.02, in Baltimore) and positive syphilis serology (OR = 4.4; *p* = 0.01 in Baltimore and OR = 3.9; *p* = 0.02 in New Orleans) were associated with confirmed HTLV-1/2 infection (37). This association persisted after adjusting for HIV status and was more robust for men (OR = 8.5; 95% CI: 3.1–23.6) than for women (OR= 3.1; 95% CI: 0.9–10.5) (37). The frequency of *T. pallidum* antibodies was also higher among HTLV-1 positive blood donors from Guadeloupe, French West Indies, than in seronegative HTLV-1 donors (10.5 vs. 1.7%, *p* < 0.001; OR= 7.01; 95% CI: 2.33–23.11; Adjusted OR = 2.83; 95% CI: 0.62–12.9) (38). An association between seropositivity for syphilis and HTLV-1 infection was observed in the general population in Gabon (19/106 vs. 83/1,134, OR = 2.77, 95% CI 1.6–4.77) (39) and Brazil (12/51 vs. 33/3,400, OR = 31.4, 95% CI 15.1–65.3) (40). In Japanese women attending antenatal care no association was found between syphilis and HTLV-1 infection (OR = 0.48, 95% CI 0.49) (41). In Haitian pregnant women although the prevalence of positive syphilis serology was higher in HTLV-1 infected women this was not statistically significant [6/44 (14%) vs. 7/89 (8%), *p* = 0.22] (42), while in Brazil an association was reported in parturient women (OR = 2.48, 95% CI 0.31–20.01) (43). In Peruvian women (sex workers and those attending antenatal care) positive serology for syphilis was also associated with HTLV-1 infection (OR = 1.78; 95% CI: 1.05–3.04, *p* = 0.03) and the prevalence of HTLV-1 infection was higher in patients seropositive for syphilis than in seronegative patients (30.3 vs. 10.2%, *p* < 0.0001) (44). Another study of female sex workers in Peru, confirmed that HTLV-1 infection was associated with history of syphilis (OR = 2.3, 95% CI 0.9–5.9) and positive syphilis serology (OR = 1.5, 95% CI 0.6–3.6). The later association did not persist after adjusting for condom use and duration of prostitution (AOR = 0.8, 95% CI 0.3–2.2) (45). In a STD clinic in Rome, Italy, 9/1,457 patients with at-risk sexual behavior were found to be HTLV-1 seropositive and the presence of antibodies to *T. pallidum* was associated with risk of HTLV-1 infection (OR = 15.62; 95% CI: 3.2–75.6 and Adjusted OR = 9.52; 95% CI: 1.7–52.3) (46). In Zaire, the OR for HTLV-1 seropositivity in syphilis co-infected sex workers was 2.8 (95% CI: 1.7–4.7), [15.2% (28/184) 15.2 vs. 5.9% (58/977)] (47). Two studies found no association between HTLV-1 infection and evidence of syphilis past or present: a study of police officers from Guinea-Bissau where no association was found between HTLV-1/2 infection and history of syphilis evaluated by *T. pallidum* hemagglutination test (48), nor in 100 HTLV-1 positive Japanese pregnant women compared to 100 uninfected controls [OR = 0.5 (95% CI: 0–5.5), *p* > 0.05] (41). Meta-analysis shows an association between syphilis and HTLV-1 co-infection, with a pooled OR ranging from 1.6 to 6.99, according to the population being considered (Figure 3). Whether *T. pallidum* seropositivity is associated with mucosal disruption and increased risk of HTLV-1 infection or is only a surrogate marker of unsafe sexual activity is unclear.

**Figure 4:**
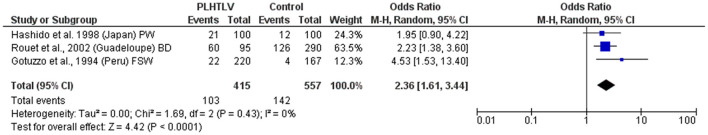
Forest Plot and meta-analysis showing the Odds Ratio *Chlamydia trachomatis* infection in people living with HTLV-1. The boxes and lines indicate the odds ratios (ORs) and their confidence intervals (CIs) for each study. The pooled odds ratio is represented by a black diamond. The size of the blue squares indicates the relative weight of each estimate. Statistical analysis and graph were performed using RevMan 5 Software. Odds Ratio was calculated using Random effects model and Mantel-Haenszel statistical method. Measures of heterogeneity between studies is shown. PLHTLV, people living with HTLV; BD, blood donors; PW, pregnant women; FSW, female sex workers.

An association has also been observed between HTLV-1 and seropositivity for *Chlamydia trachomatis (C. trachomatis)* amongst Peruvian female sex workers (with an OR = 3.7; 95% CI 1.4–13.2 after adjusting for condom use) (45). This association was previously reported for sex workers and pregnant women from Ivory Coast (16) and in a case control study in Guadeloupean blood donors where the detection of antibodies to *C. trachomatis* was higher in cases [63.2% (60/102)] than controls [43.4% (126/306), *p* < 0.001, OR = 2.23; 95% CI 1.38–3.61; Adjusted OR = 1.95; 95% CI 1.03–3.68] (38). Although the prevalence of antibodies to *C. trachomatis* was also higher in HTLV-1 seropositive pregnant women from Japan, the difference was not statistically significant (21/100 vs. 12/100; OR = 1.9; 95% CI 0.8–4.2; *p* > 0.05) (41). When these studies are analyzed altogether it is observed that PLHTLV are 2.4 times more likely to be infected by *C. trachomatis* than those without HTLV-1 (Figure 4). Zunt et al. analyzed the cervical shedding of HTLV-1 by detecting HTLV-1 proviral DNA in cervical samples collected from Peruvian sex workers. They observed that cervicitis (characterized by increased number of polymorphonuclear cells in cervical mucus) increased the cervical shedding of HTLV-1, but not *Chlamydia* or Gonorrhea (OR = 1.5; 95% CI 0.6–3.8 and OR=1.1; 95% CI 0.2–5.2, respectively). As the authors highlighted, the limited number of co-infected individuals may have influenced the results (49).

Human T lymphotropic virus type 1 infection was associated with HSV-2 seropositivity in pregnant women from Haiti (OR = 3.7, 95% CI 1.6–11.5) (42) and female sex workers from Peru [Adjusted OR (condom use and duration of prostitution) = 3.7, 95% CI 0.5–28.4] (45). The authors hypothesized that HSV-2 may lead to disruption of the mucous membrane increasing susceptibility to sexual transmission of HTLV-1 (42). Changes in the frequency of immune cells at the mucosa, including HTLV-1 target cells (lymphocytes) or transporters (dendritic cells) as well as the expression of activation markers in lymphocytes are factors that may influence susceptibility to HTLV-1 infection.

**Figure 5:**
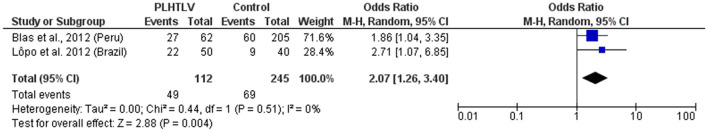
Forest Plot and meta-analysis showing the Odds Ratio HPV infection in people living with HTLV-1. The boxes and lines indicate the odds ratios (ORs) and their confidence intervals (CIs) for each study. The pooled odds ratio is represented by a black diamond. The size of the blue squares indicates the relative weight of each estimate. Statistical analysis and graph were performed using RevMan 5 Software. Odds Ratio was calculated using Random effects model and Mantel-Haenszel statistical method. Measures of heterogeneity between studies is shown.

Human T lymphotropic virus type 1 and HPV are both sexually transmitted oncogenic viruses that cause persistent infection. The progression to cervical cancer due to HPV is more frequent among immunosuppressed women, such as those co-infected with HIV. Studies concerning HTLV-1/HPV co-infection are limited. In Brazil, the prevalence of HPV infection was higher among HTLV-1 infected (mean age 38 years) than uninfected women (mean age 36 years) attending a gynecology clinic (44 vs. 22.5%, *p* = 0.03) (50). Human T lymphotropic virus type 1 proviral load (PVL) was similar in HTLV-1 mono-infected and HTLV-1/HPV co-infected women, no cervical cancer and only one case of high grade squamous intraepithelial lesion was observed (50). In HTLV-1 infected women from the Peruvian Amazon the prevalence of HPV infection was also higher than in HTLV-1 negative individuals (43.6 vs. 29.3%). They were twice as likely to have HPV infection of any type (OR = 2.1 95% CI 1.53–2.87) and to have high-risk HPV infection (OR =1.93 95% CI 1.04–3.59) than those not infected by HTLV-1, after adjusting for confounding variables, including age, education, age of sexual partner, number of sexual partners within the last 12 months, and condom use at last sexual intercourse (51). However, no difference was found between the prevalence of epithelial cell abnormalities in the cervix or presence of low-grade squamous intraepithelial lesion (LSIL) or higher grade lesion, according to HTLV-1 infection status (51). Figure 5 shows the forest plot and meta-analysis regarding the association of HPV and HTLV-1 infection.

There is a consistency in the literature to indicate a relationship between HTLV-1 and coinfection with other sexually transmitted infections (STIs) [Pooled OR (95% CI): Syphilis = 4.6 (2.4–8.8); Chlamydia = 2.36 (1.6–3.4); HPV = 2.07 (1.3–3.4)]. However, causation has not been demonstrated. Prospective studies are required and would be helpful to distinguish between HTLV-1 infection acquired sexually and vertically.

### Hepatitis

**Figure 6:**
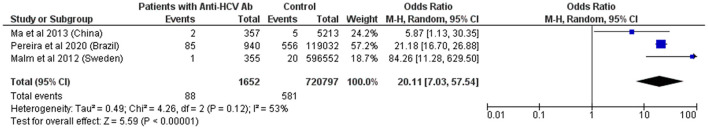
Forest Plot and meta-analysis showing the Odds Ratio HTLV-1 infection in individuals with anti-HCV antibodies. The boxes and lines indicate the odds ratios (ORs) and their confidence intervals (CIs) for each study. The pooled odds ratio is represented by a black diamond. The size of the blue squares indicates the relative weight of each estimate. Statistical analysis and graph were performed using RevMan 5 Software. Odds Ratio was calculated using Random effects model and Mantel-Haenszel statistical method. Measures of heterogeneity between studies is shown.

Several studies report a high frequency of HCV and HBV infection in PLHTLV. Unfortunately, most of them do not include a control group. Studies from China, Brazil, and Sweden confirmed a higher prevalence of HTLV-1 infection among patients that are seropositive for HCV (52–54). Pooled OR revealed that individuals with anti-HCV antibodies are 20 times more likely to be infected with HTLV-1 (Figure 6).

### Respiratory Infections

Human T lymphotropic virus type 1 can cause pulmonary disease that may result in important parenchymal damage and bronchiectasis (55). However, little is known about HTLV-1 and respiratory co-infections. A study conducted in Australia revealed an association between HTLV-1 and lower respiratory tract infection, after adjusting for covariates (adjusted negative binomial regression, coefficient 0.19; 95% CI, 0.04–0.34) (56). It would also be interesting to know the impact of HTLV-1 and SARS-CoV-2 coinfection, but no data are available yet.

## Does HTLV-1 Infection Increase the Probability of the Persistence of a Co-existing Pathogen?

Human T lymphotropic virus type 1 infection may also influence the ability of other parasites to persist within the co-infected host. This was described in HTLV-1 and *St. stercoralis* co-infection. Patients with HTLV-1 have more frequently positive stool test for Ss and treatment failure (persistent parasite excretion despite treatment). This topic is addressed in another study.

**Figure 7:**
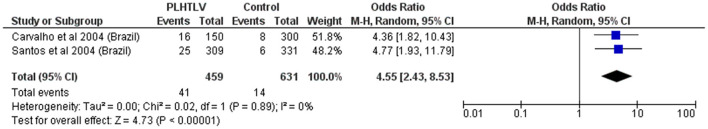
Forest Plot and meta-analysis showing the Odds Ratio *Schistosoma mansoni* infection in people living with HTLV-1. The boxes and lines indicate the odds ratios (ORs) and their confidence intervals (CIs) for each study. The pooled odds ratio is represented by a black diamond. The size of the blue squares indicates the relative weight of each estimate. Statistical analysis and graph were performed using RevMan 5 Software. Odds Ratio was calculated using Random effects model and Mantel-Haenszel statistical method. Measures of heterogeneity between studies is shown.

Similarly, high frequency of *Schistosoma mansoni* infection among HTLV-1 infected individuals from Brazil [26/309 (8.4%) vs. 6/331 (1.8%) controls, *p* = 0.0003] has been reported (57). Two available studies combined give an OR = 4.6 (*p* < 0.001), but both were carried out in Brazil by the same group, and probably have an overlap of patients (Figure 7). Treatment failure, defined as the persistence of *S. mansoni* eggs in stool samples 2 months after therapy with praziquantel was more frequent in HTLV-1 co-infected patients than in patients with schistosomiasis only [4/20 (20%) vs. 1/44 (2.3%), *p* < 0.05]. Re-exposure to parasite was excluded in all patients. One co-infected patient was not able to eradicate the helminthic infection even after eight courses of praziquantel (58). Although limited, evidence points to therapeutic failure and persistence of *Schistosoma mansoni* in people living with HTLV-1 (PLHTLV) but this is better characterized for *Strongyloides* infection.

## Does HTLV-1 Impact the Pathology of Co-infection?

Unlike HTLV-1, *Schistosoma* sp infection elicits a Th-2 type response. Although it seems to be important for parasite clearing, it can be associated with the disease‘s pathophysiology (5). High degree of infection associated with host's immune reaction to parasite can lead to granuloma formation and consequently liver fibrosis, which is the most important pathological finding and occurs in up to 5% of chronic infected individuals. Thus, HTLV-1/*S. mansoni* co-infection may result in some beneficial effect to the host. Indeed, the number of eggs/gram of stool was lower in co-infected patients (58) and an inverse association was found between HTLV-1 infection and liver fibrosis in patients with schistosomiasis (evaluated by ultrasound in 22 co-infected patients and 40 mono-infected with *S. mansoni*) (57, 58). However, Goon and Bangham (5) hypothesized that lower rates of egg excretion observed in the stool of co-infected individuals may be associated with either lower levels of IL-4 (as this cytokine seems to be necessary to translocate eggs from mesenteric venules through intestinal wall followed by secretion in animal models), or with an impaired control of *Schistomosoma*'s reproduction which could result in egg excretion even in individuals with low parasite burden. As discussed before, persistence of *S. mansoni* eggs in stool samples after therapy is more frequent in HTLV-1 co-infected patients than in patients with schistosomiasis only (58).

Regarding TB severity in HTLV-1 infected individuals, the findings are contradictory. In Peru, HTLV-1 patients co-infected with TB reported more frequently a history of relatives‘ deaths due to TB infection (25). Human T lymphotropic virus type 1 co-infected patients also were more likely to have a highly positive sputum smear (more than 10 acid-fast bacilli in at least 20 fields in microscopy stained by Ziehl-Neelsen) than those mono-infected individuals (59), indicating that HTLV-1 patients may have an impaired ability to control bacilli replication. This finding is reinforced by the decreased skin reaction to tuberculin skin test (TST) observed in co-infected HTLV-1/TB patients and HTLV-1 mono-infected asymptomatic carriers (60–62). Human T lymphotropic virus type 1/HIV-2 co-infected patients hospitalized for TB have a higher CD4 count and higher mortality rate when comparing to HIV-TB infected subjects from Guinea-Bissau (15). Pedral-Sampaio et al. showed an increase in mortality in co-infected individuals: amongst HIV-1 and HTLV-1 negative TB patients the mortality was lower (8%, 25/319) than in patients with HTLV-1 (25%, 8/32) or HIV-1 (33%, 6/18) co-infections. Five out of nine (56%) patients with triple infection (TB-HIV-1-HTLV-1) died (21). On the other hand, in another study from Brazil (Bahia), no differences were observed in clinical presentation of TB according to HTLV-1 status, nor in the number of hospitalizations, sputum and TST response, however the number of patients was small (13 HTLV-1/TB and 24 TB mono-infected), they all had severe active TB (as they were all hospitalized) and most were malnourished, which may have impacted the findings (63).

A study from the Democratic Republic of Congo analyzed 378 leprosy patients for 22 years and reported a significant decrease in the survival of *M. leprae*/HTLV-1 co-infected patients: Mortality rates 5.5/100 vs. 3.6/100 person-years of observation, resulting in a risk ratio of 1.4 (95% CI 1.04–1.89) for those infected with HTLV-1 (28). In a study of the impact of viral co-infections (including HTLV-1, HIV, HBV, and HCV) on leprosy outcome in Brazil, co-infected patients showed higher rates of neuritis, nerve function impairment and relapse, but those viruses were analyzed altogether (34). A study from Japan and another one conducted at Congo and Ivory Coast, did not observe differences among HTLV-1 prevalence between patients with lepromatous and tuberculoid forms of leprosy (27, 30).

The impact of HTLV-1 on HCV infection is still debatable. Most studies report a deleterious impact on HCV outcome and disease severity on co-infected patients. Higher HCV viral load (64, 65), higher IFN-γ serum concentration (66), higher degree of liver steatosis (66), higher risk of hepatocellular carcinoma (67), reduced HCV clearance (both natural and in response to IFN treatment) (65, 68, 69), and decreased survival rates after HCV-related liver transplantation were already reported in HCV/HTLV-1 co-infected patients comparing to HCV mono-infected individuals (70, 71). A study from Miyazaki, Japan, showed that HTLV-1 co-infection increased the risk of self-reported incident liver disease (RR = 3.5, 95% CI 1.9–6.4) and registered death due to liver cancer (RR = 8.2, 95% CI 1.6–441.4) (72). Human T lymphotropic virus type 1 Tax protein also facilitates HCV replication *in vitro* (73). On the other hand, HTLV-1/HCV patients were reported to have lower levels of markers of hepatic damage (ALT, AST, GGT, bilirubin) (74–76) and increased platelet counts (74). No difference between HTLV-1 PVL and HCV viral load between co-infected patients and mono-infected individuals were reported by others from Brazil (74).

The association between syphilis and HTLV-1 has been described above. However, there are no data concerning the impact of this coinfection on each disease outcome. There is just one case report of atypical long-lasting secondary syphilis with a localized and atypical eruption in a HTLV-1 co-infected individual (77).

Crusted scabies (also called Norwegian scabies) is an infrequent, albeit severe and highly contagious infection caused by massive infestation of the mite, *Sarcoptes scabiei* (*S. scabiei*) and may be considered a marker of HTLV-1 infection (78). In Bahia, Brazil, severe scabies (more than 80% of body surface affected but not fulfilling the crusted scabies criteria) was associated with HTLV-1 infection (OR = 3.0; 95% CI 1.85–4.86, *p* < 0.01) (79). History of chronic scabies were also associated with HTLV-1 infection in Peruvian women (OR = 13, 95% CI = 1.6–82, *p* < 0.02) (80). In Peru, in two different studies, 60–70% of patients with crusted scabies were infected with HTLV-1 (81, 82), while in two case series all six patients in French Guiana (83) and all 21 patients from Brazil were co-infected with HTLV-1 (79). Among HTLV-1 seropositive hospitalized patients in Dominica, 7.6% had crusted scabies (84). In the indigenous Australian population scabies was more frequent in HTLV-1 infected patients than seronegative individuals [14.2% (72/507) vs. 8.5% (80/944), *p* < 0.001] (56). Crusted scabies was already reported in patients with ATL (82, 83, 85–87), with HAM/TSP (81, 82, 88) and in asymptomatic individuals co-infected with other infectious agents (Ss and TB) or other comorbidities (82, 83, 89, 90). During 30 months' follow-up of 30 children with HTLV-1 associated infective dermatitis (ID) in Brazil, 70% had scabies, and one had crusted scabies (91). Therefore, HTLV-1 may be associated with severe cases of scabies, a clinical marker of immunosuppression.

Fungal infections are frequently considered opportunistic infections and may be used as a warning sign of immunological impairment. The prevalence of HTLV-1 infection was higher in Japan among patients with pulmonary cryptococcosis than controls with other pulmonary disorders [32.6% (6/19) vs. 13.8% (49/356), *p* = 0.033] (9). Dissemination of *C. neoformans* to the CNS was not observed regardless of HTLV-1 status (9). Cryptococcal lymphadenitis was reported in one HTLV-1 individual in a small case series including two patients with HIV-1. The HTLV-1/cryptococcus infected patient had a smaller number of fungal cocci in the focal area and a higher CD4^+^ cell count compared to the two HIV/*Cryptococcus* co-infected individuals (92). Disseminated *Cryptococcus* infection with CNS involvement was described in a HTLV-1 patient from the Caribbean (93). Pulmonary cryptococcosis in a HTLV-1 positive, HIV-negative patient with disseminated molluscum contagiosum was also described (84). Cryptococcosis has been reported in patients with ATL (94–96).

*Histoplasma capsulatum* is a ubiquitous fungus that usually causes asymptomatic infection. Patients co-infected with HIV, those with primary immunodeficiencies or under immunosuppressive treatments are at risk of developing progressive disseminated histoplasmosis (PDH), a clinical illness with extra-pulmonary involvement. The mortality of PDH can be as high as 85% in untreated patients, and 25% despite a standard “adequate” therapeutic regimen. Severe gastrointestinal histoplasmosis in Peru (97), spinal cord histoplasmoma (98) brain granulomas and lymphoma associated with several other opportunistic infections (99) have been reported in HTLV-1 co-infected individuals, but no conclusive evidence of an association between HTLV-1 and histoplasmosis outcome was provided (100).

*Paracoccidioides brasiliensis* is a dimorphic fungus restricted to Latin America. It is the agent of paracoccidioidomicosis, a systemic fungal disease. Four cases of HTLV-1/*P. brasiliensis* co-infection seen during a 2 years-period in a Peruvian hospital were reported. Two had severe chronic clinical presentation and two had aberrant clinical picture and co-infections (one co-infected with *H. capsulatum* and the other had sepsis caused by *Listeria monocytogenes*) (101).

The strongest evidence points to increased pathology due to mycobacterium in the presence of HTLV-1. There is a suggestion that HTLV-1 infection may favorably modulate the impact on *S mansoni* but this needs to be substantiated. Whilst it is plausible that HTLV-1 infection may impact the course of the many mycotic infections that co-exist in HTLV-1 endemic areas the need for better studies to determine the epidemiological and biological associations between HTLV-1 and infections such as histoplasmosis, paracoccidioidomycosis, and cryptococcosis is clear (100).

## Does Co-infection Impact Pathology of HTLV-1?

Data concerning the impact of co-infections in HTLV-1 outcome is scarce. It is known that *Schistosoma* antigens downregulate *in vitro* pro-inflammatory cytokine response (IFN-γ and CXCL9) by peripheral blood lymphocytes from HTLV-1 infected individuals (102, 103). These antigens also upregulate IL-10 production (103). Interestingly, high prevalence of helminthic infection (*Schistosomo mansoni* and *St. stercoralis*) was observed among asymptomatic carriers when compared to individuals with HAM [23% (71/310) vs. 3% (1/32), *p* < 0.05] (104). Human T lymphotropic virus type 1 PVL was lower in co-infected patients [2.2 copies/100 cells vs. 3.7 copies/100 cells, *p* < 0.05]. They also showed lower levels of IFNg, CD8+IFN+ (57, 104), CD4+IFNg+, and an increase in T cells expressing IL-5 and IL-10 compared to HTLV-1 monoinfected patients (104). These observations, together with the immunological findings gave rise to the hypothesis that HTLV-1/helminthic co-infection in comparison to HTLV-1 single infection may decrease Th1 response, which may influence the clinical outcome of HTLV-1 infection (104). As the majority of the data were obtained by evaluating patients with HTLV-1/*St. stercoralis* and HTLV-1/*Schistosoma* sp. together, more studies are needed to clarify the impact of *Schistosoma* sp. on HTLV-1 clinical outcomes.

Regarding TB and HTLV-1 infection outcome, a history of TB was associated with an increased risk of HAM development among HTLV-1 infected individuals in Brazil (OR 3.8; 95% CI 1.9–9.1, *p* = 0.0001) in a retrospective association (105). Once again, changes in immunological response may be responsible for the observed alteration. Among patients with HAM, IFN-γ/IL-10, and TNF-α/IL-10 ratios were higher in those individuals with active TB than those HAM patients without co-infection. These differences were not observed in HTLV asymptomatic carriers nor in those with probable HAM (105). However, no differences in HTLV-1 PVL and serum CCXCL9 and CXCL10 levels according to TB infection status (not infected, latent infection, and active infection) were observed among patients with HAM. These findings led to the hypothesis that the higher susceptibility to develop TB in HTLV-1 infected individuals may be due to an impaired innate immune response (such as lower levels of TNF-α). However, more severe clinical presentation observed in co-infection may be due to an enhancement of Th1 response. Thus, the co-infection TB/HTLV-1 could increase not only TB severity, but also HAM incidence. More studies regarding HTLV-1 associated diseases in patients co-infected with TB are necessary.

A recent study amongst the Indigenous population of Central Australia, demonstrated that although HBV co-infection did not impact HTLV-1 PVL, HBV was associated with increased expansion of HTLV-1 T-cell infected clones. Moreover, the degree of clonal expansion positively correlated with the titer of HBsAg. This finding indicates that HBV/HTLV co-infection may have a role in the development of ATL (106) and this study should be replicated for other infections. Although clinical data to support this hypothesis are still limited, chronic HBV co-infection was found in 47% (8/17) and chronic HCV in 35% (6/17) of patients with ATL in a case series from Taiwan (compared to 15–20 and 2% prevalence found in general population) (107). Hepatitis C virus infection was identified as a predictor of mortality in PLHTLV in Brazil [HR (95% CI) = 5.2 (2.4–11.1), *p* < 0.0001] (108). However, there are evidences that HCV co-infection does not impact HTLV-1 PVL, T-cell proliferation rate (109) nor the frequency of HAM (74).

During the follow-up (mean 6.75 years) of 36 HTLV-1 infected patients with ID from Bahia, 47.2% developed HAM and one individual developed ATL (110). In a more recent study, the same group showed that during follow-up of 37 patients with ID 54% developed HAM before the age of 19 years old (111). HTLV-1 associated myelopathy was observed in 30% of children with ID in Brazil (112), and a history of ID was more frequent in patients with HAM than in asymptomatic carriers (3/73 vs. 0/120, *p* = 0.02) (113). Moreover, in 74 ATL cases in Bahia, 44% had history of severe eczema in childhood, suggestive of ID (114, 115). Interestingly, 30% of patients with ID had abnormal lymphocyte in peripheral blood and 16.6% had flower cells (116). A number of factors may contribute to these associations between ID and HAM and ATL. First, infection in early life is strongly associated with ATL, but the young age of ATL onset must still be explained. Second, ID is associated with high HTLV-1 PVL, a risk for both HAM and ATL. Third, ID which is characterized by both lymphocytic infiltration in the skin and by persistence of, and pathology due to, bacteria, may represent both the inflammatory and the immunosuppressive consequences of HTLV-1 infection.

Data regarding the impact of co-infections on HTLV-1 outcome is extremely limited and more studies are needed.

## Conclusion

There is evidence of higher rates of HTLV-1 infection in association with other blood borne and STIs but whether this represents increased susceptibility or opportunity is unclear. The data suggest that HTLV-1 increases susceptibility to a range of infection related disease resulting in either higher risk of disease if exposed, longer persistence or greater severity. This indicates that PLHTLV may have a degree of immunosuppression regardless of symptomatic or asymptomatic HTLV-1 clinical status. Co-infections may also impact the risk of developing HTLV-1 associated disease, but data are still extremely limited.

Human T lymphotropic virus type 1 infection is a neglected infection that is responsible not only for diverse and severe inflammatory diseases, such as HAM, uveitis, pulmonary diseases, Sjogren syndrome, and arthritis, and the malignant disease ATL, but also a degree of immunosuppression that can increase susceptibility to and/or severity of co-infections, many of which are also neglected tropical diseases. More subtle impact of HTLV-1 on the response to more common infections, might contribute to the increased mortality rate of HTLV-1, but this will only be determined by large scale prospective controlled studies. Identification of HTLV-1 status should therefore be included in population-based studies. Human T lymphotropic virus type 1 should be considered a global health concern as its impact in public health is evident. Measures to improve the global awareness about HTLV-1 and to prevent its transmission are necessary.

## Author Contributions

GT designed the study, supervised the project, and revised the manuscript. CR performed data compilation, analysis, and wrote the first draft of the manuscript. All authors contributed to the article and approved the submitted version.

## Funding

GT was supported by NIHR Imperial College Trust.

## Conflict of Interest

The authors declare that the research was conducted in the absence of any commercial or financial relationships that could be construed as a potential conflict of interest.

## Publisher's Note

All claims expressed in this article are solely those of the authors and do not necessarily represent those of their affiliated organizations, or those of the publisher, the editors and the reviewers. Any product that may be evaluated in this article, or claim that may be made by its manufacturer, is not guaranteed or endorsed by the publisher.
